# A Comprehensive Study on Epidemiology Case Studies Using Computational Analysis

**DOI:** 10.1155/2022/6508866

**Published:** 2022-09-07

**Authors:** Xinjiang Lin, Shouping Chen, Amatul Bushra Akhi

**Affiliations:** ^1^Annual Ring Orthopaedic Hospital Group, Changsha, Hunan 410000, China; ^2^Medical Management Center of Annulus Orthopaedic Hospital Group, Hunan 410000, China; ^3^Department of CSE, Daffodil International University, Ashulia, Dhaka, Bangladesh

## Abstract

Health-related issues and occurrences with regard to a particular population are the subject of an epidemiology study. This paper presents the results of a retrospective epidemiological investigation on 15922 hospitalized hand trauma patients from Central China between 2011 and 2020. Gender, age, onset season, injury mechanism, injury environment, injury location, and clinical characteristics are among the characteristics of the data gathered. The study is using computational analysis to draw inferences from the case studies collected in the databases of the hospitals. The types and characteristics of occupational injuries at home and outdoor are compared and analyzed. The purpose of the study is to present the findings from recent case studies for future reference and to recommend useful roles for the industrial sector in the care of patients with hand trauma in order to lower occupational harm. The injuries of preschool children are also analyzed. The incidence rate of hand injuries in infants has been increasing year by year which is directly related to the inefficient growth of children in rural areas. The data are collected from hospitals, then the data analytical tools are applied to draw conclusions. The suggested model is intelligently learned through the application of computational techniques, which are also used to suggest treatments to trauma victims. According to this study, males are more likely than females to sustain hand trauma; occupational injuries are more common than living injuries; males between the ages of 20 and 50 are at an increased risk of suffering an occupational injury. This study showed that the proportion of hand trauma in preschool children was higher (12.27%), and the 2-3-year-old group was the main injury target of preschool children (45.70%). The accidental injuries of newborns and young children can be reduced by government assistance, social support, and tighter monitoring.

## 1. Introduction

Human creation, life, and aesthetics all heavily rely on the hand. It is regarded as one of the most vital organs, second only to the face, and one of the body's most delicate portions. Thirty years ago, in [1] the authors analyzed 50,272 cases of hand trauma and found that 26% of them were injured at work, 34% were injured at home, and 15% were injured by participating in sports. The study's findings also demonstrate that various situations, including the workplace, the home, sports, and auto accidents, have their own unique internal laws that apply to hand injuries. Separate analysis and research are required. Twenty years ago, Jishuitan Hospital in China country reported that hand injuries accounted for 28.6% of emergency trauma patients [2]. In the past 10 years, research in China has shown that occupational hand trauma patients account for 50%–80% of the total number of hand trauma, and it is the most common occupational injury in the emergency department. Hand injuries account for about 37% of all occupational injuries. The incidence of hand trauma has significantly increased, primarily because of the quick growth of small private businesses and the relatively antiquated production equipment, as well as the lack of focus on safety facilities and safety education, unfamiliarity with the use of machines, and job changes. Factors such as temporary overtime and not wearing protective equipment have led to an increase in the incidence of occupational hand injuries year by year. We retrospectively analyzed the epidemiological data of hand trauma in a significant orthopaedic hospital in Central China over the previous ten years. In order to study the epidemiological characteristics of hand trauma there, determine the regularity of its incidence, and provide a basis for its prevention and treatment.

Located in Central China, Changsha covers an area of 11,819 square kilometers and has a population of more than 10 million. It is an important provincial capital, a national historical and cultural city, a city with the best international image, and a leisure and entertainment city with a high happiness index. It is also a Chinese project. The capital of machinery, industrial manufacturing, construction, and handicrafts are also developed.

There are 30 large-scale tertiary hospitals in Changsha, and the medical and health services are well developed. The Annual Ring Orthopedic Hospital Group is a large-scale orthopaedic specialized hospital chain nationwide, with strong specialized technology, especially hand surgery technology. Therefore, more than 2,000 cases of hand trauma patients are received every year. In addition to the province as a whole, these patients originate from the city. From rural areas, road traffic, industry, and building sites, as well as from offices, service facilities, and schools. We believe that collecting and analyzing the epidemiological data of hand trauma patients in this hospital in the past 10 years is valuable for understanding and mastering the modern epidemiological history of the disease, the regularity of incidence, clinical characteristics, and assisting the government in formulating preventive measures to reduce the incidence.

The hands and digits of the human being occupy a major role in our professional life. Thus, not surprisingly, hand injuries are the most frequent bodily traumas sustained at work [3]. Even seemingly minor injuries can produce a devastating functional loss. Accurate, early diagnosis and definitive treatment are essential to minimize the functional loss and avoid the socioeconomic effects of protracted disability. Hand injuries due to occupational accidents or work-related hand injuries (WRHIs) lead to more serious consequences than any other organs in terms of both clinical courses and economic losses [4]. These injuries are as preventable as the other bodily traumas [5].

At least a million people are admitted to the emergency departments (ED) due to acute WRHI in the USA each year [6]. The incidence is particularly high in industries and jobs in which hand intensive work is done [5]. Hand trauma is a common surgical disease, and with the development of industrial machinery production, the incidence of acute occupational hand trauma is increasing [7]. Data show that acute occupational hand injuries account for 36.3% to 76.9% of emergency hand injuries [8], and Jishuitan Hospital in China reported that hand injuries account for 28.6% of trauma emergency patients [2]. In [9], the authors reported 732 cases of occupational hand trauma patients, the incidence of males was more than that of females. The epidemiological characteristics of occupational hand trauma patients reported by [10–12] are similar to the data in this study.

Among those with hand trauma, 76% were American men; 69% were British men; 59% were Danish men; 83% were Turkish men. In the Netherlands and Denmark, the age group between 10 and 14 year-old has the highest incidence. 44% of patients in Canada are 10–19 years old; 35% of patients in Turkey are under the age of 15 [13–16]. Among the occupational injuries in the statistics of the US labor department, hand trauma accounts for the largest proportion, accounting for 30% to 37% of all occupational injuries [17]. In [18], studies have shown that there is a statistical relationship between hand trauma and social deprivation, which varies with the type of injury.

Occupational injuries cannot be disregarded given China's tremendous advancement in science and technology, economy, and industrial manufacture. At present, China still lacks comprehensive and systematic big data analysis of hand trauma epidemiology data in the past ten years. This paper comprehensively and systematically analyzes and studies the distribution characteristics, injury mechanism, and clinical characteristics of hand trauma in different groups of people and provides a scientific basis for the government and enterprises to make decisions on the prevention and control of hand trauma.

Hand trauma is the most common trauma in people's production and life. According to literature reports, in emergency trauma cases, the proportion of hand trauma is between 15% and 28.6% [1, 19]. Reference [20]. So this study analyzes the percentage of hand trauma, patient's gender, age, onset season, injury mechanism, injury environment, injury site, and its clinical characteristics. The main highlights of the paper are as follows:

1. Collect and analyze the data of 15,922 patients who met the criteria for hand trauma among inpatients from 2011 to 2020.
2. To study the composition and distribution of hand trauma.
3. To study the risk factors of hand trauma.
4. Factors such as gender, age, the season in which the injury first occurs, the mechanism by which the damage occurred, the environment in which it occurred, the location of the injury, and its clinical characteristics are considered. The data are evaluated for preschoolers' injuries, life injuries, and occupational injuries, and compare them to domestic, Longitudinal, and horizontal comparative studies.
5. Research on preventive measures of hand trauma is conducted.

Rest of the paper is organized as follows: in Section 1, background details of epidemiology hand trauma incidents and state of art of the epidemiology case studies are discussed. In Section 2, the proposed method of data collection, inclusion, and exclusion criteria of patients are discussed. In Section 3, result analysis and observations are discussed followed by a conclusion and references.

## 2. Proposed Method

### 2.1. Data Recording and Coding

Data from the Annual Ring Hospital Information System (HIS) is considered, which included nine variables: admission date, month, and year; Sex; Age code; Age group; Injury caused; Product code and location code. The cause of injury was coded by International Classification of Diseases Ninth Edition (ICD-10).

### 2.2. Inclusion Criteria

1. All injuries of the wrist joint and beyond; soft tissue injuries of upper limbs near wrist joints, such as nerve, tendon, blood vessel injuries, skin and soft tissue injuries in a large area, etc., but excluding injuries and burns of bones and joints within this range.
2. The visit time was within 72 hours of injury.
3. The medical records are complete.

### 2.3. Exclusion Criteria

1. Chronic old hand injury.
2. Hand diseases and deformities.
3. Incomplete medical records.

### 2.4. Research Method

There are 3 wards in the Hand Surgery Department of the Annual Ring Orthopedic Hospital, which consists of 15 physicians and 30 nurses, including 1 chief physician and 3 deputy chief physicians. Each ward has a part-time secretary. The director and secretary of the department are responsible for the quality of medical records and are required to fill in each medical record in a timely and complete manner, including the patient's basic information, injury, treatment, surgical methods, and recovery status and upload the HIS.

The previous 10-year medical records of hand trauma patients in the department were collected and sorted by the secretary of the department, and those who met the inclusion criteria were counted. In order to ensure the accuracy of the data, the hospital medical record room reviews the data compiled by each department and corrects the wrong information. The statistical contents include the following:

1. General information (sex, age, occupation, etc.).
2. Injury conditions (environment, nature, location, cause, etc.).
3. Specific tissue damage (location and type, etc.).
4. Treatment status (hospitalization time and outcome, etc.).
5. Infants and young children morbidity, etc.

### 2.5. Statistical Analysis

To estimate the frequency and percentage of all reported injuries, to describe the incidents, to specify the characteristics of injury brought on by consumer products, and to examine the risk factors, geographic locations, and seasonal patterns of injuries, descriptive epidemiological methods were used. SPSS 20.0 statistical software was used for analysis. The counting data was represented by the constituent ratio (%), and x^2^ test was used for comparison between samples. Measurement data were expressed as *X* ± *s*, and *t*-test was used for comparison between samples. *P* < 0.05 was considered statistically significant. Examine the injury's origin, location, season, type, and any possible correlations with age and sex. Age groups were analyzed according to six levels (0–6, 7–14, 15–18, 19–30, 31–65, and 66+).

## 3. Results and Analysis

### 3.1. Results

#### 3.1.1. General Situation

**Figure 1:**
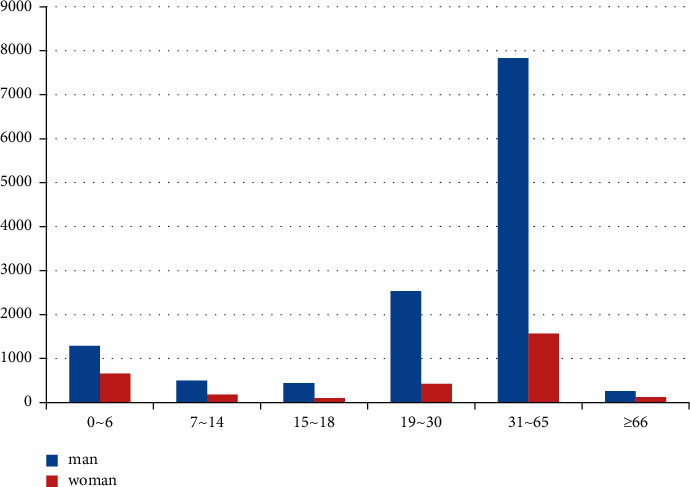
Distribution of age and sex. (*x*2 = 22489.14, *P* = 0.001).

15922 inpatients with hand trauma were analyzed. Among them, 12897 cases were male (81.00%), 3025 cases were female (19.00%), and the ratio of male to female was 4.26 : 1. The incidence rate of the male was higher than that of females, and the difference was statistically significant (*x*^2^ = 6120.86, *P* = 0.001). The oldest is 93 years old and the youngest is 5 days old. The mean age was (34.97 ± 18.23) years, including (*x* = 35.71 ± 17.50) years for males and (*x* = 31.81 ± 20.77) years for females. The gender distribution of each age group as shown in Figure 1 and is statistically significant. Among them, 49.21% were young and middle-aged patients aged from 31 to 65. Preschool children aged 0–6 years accounted for 12.27%.

**Figure 2:**
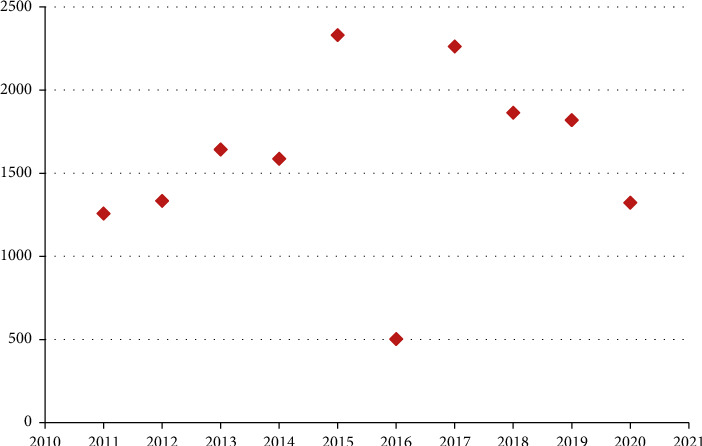
Distribution of the number of patients in each year (*x*2 = 1607.94, *P* = 0.001).

**Figure 3:**
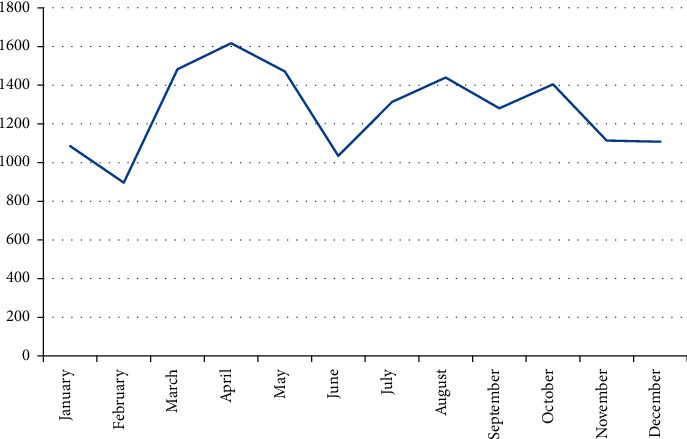
Distribution of morbidity in each month (*x*2 = 555.05, *P* = 0.001).

Figures 2 and 3 depicts the annual and monthly distribution of in patients. The lowest is in February (5.63%) and the highest is in April (10.16%) every year.

#### 3.1.2. Nature of Injury

The main injuries were occupational injuries, with 10,866 cases (68.25%) and 5,056 cases (31.75%) of life injuries. Among the occupational injury patients, 9121 cases were male (83.94%), 1745 cases were female (16.06%), and the ratio of male to female was 5.23 : 1. The age was (*x* = 40.93 ± 16.28) years old, among which the age group was mainly from 20 to 50 years old, accounting for 74.18%.

There are two types of injured environments: indoor and outdoor. Indoor: factories, shopping malls, service places, leisure and entertainment places, office places, families, etc., are considered. For outdoor environment construction sites, roads, sports venues, fields, mountains, etc. According to their occupational injuries and life injuries, the patients in this group are different. Tables 1 and 2 shows the distribution of the injured environment and injured site.

#### 3.1.3. Injured Area

**Figure 4:**
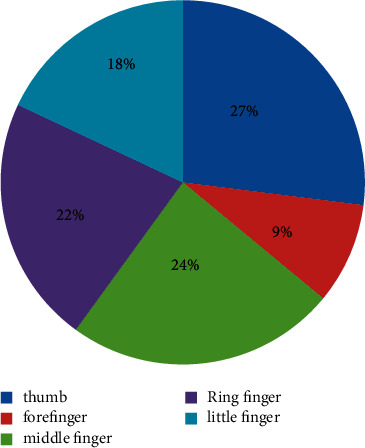
Distribution of finger injuries (*x*2 = 785.87, *P* = 0.001).

In this group, 8437 cases (52.99%) were injured in the left hand, 7244 cases (45.50%) in the right hand, and 241 cases (1.51%) in both hands. Among them, there were 6687 finger injuries, with a total of 7757 fingers. The distribution of finger injuries is shown in Figure 4. There were 162 finger injuries and 369 finger injuries with one hand. 3651 fingers were injured in the left finger and 3036 fingers were injured in the right finger. The ratio of left finger injury to right finger injury was 1.2 : 1.

There are different types of occupational injuries and life injuries, as shown in Table 3.

#### 3.1.4. Morbidity in Preschool Children

**Figure 5:**
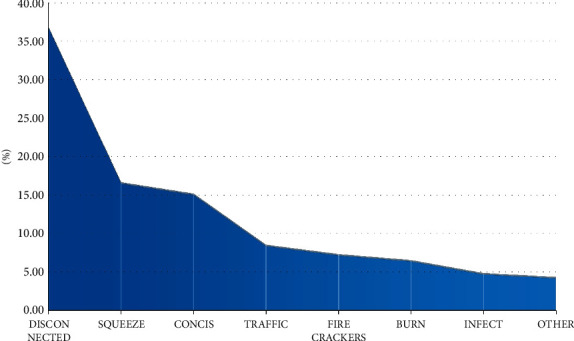
Causes of injuries of preschool children (*x*2 = 1286.049, *P* = 0.001).

There were 1954 preschool children aged 0 ∼ 6 years (12.27%), including 1293 males and 661 females. Male: female is 1.96 : 1. Among them, there were 373 infants under 1-year-old (19.09%), 893 infants aged 2–3 years old (45.70%), and 688 children aged 4–6 years old (35.21%). The youngest is 6 days, because the baby is tied with ropes when wearing clothes, which causes strangulation and infection of the skin of both upper arms. The most common cause of injury was broken fingers (36.85%), as shown in Figure 5.

#### 3.1.5. Hospital Stay

The length of hospital stay was (*x* = 11.22 ± 12.75) days. 48.03% were hospitalized within 7 days. 8–14 days accounted for 31.38%; 15–21 days accounted for 12.15%; ≥22 days accounted for 8.44%, and the longest was 111 days. The length of stay was significantly different (*x*^2^ = 6235.319, *P* = 0.001).

#### 3.1.6. Treatment Outcome

903 cases (5.67%) were cured clinically. 15011 cases (94.28%) improved; 8 cases (0.05%) were not cured. There was a significant difference in treatment outcome (*x*^2^ = 26688.02, *P* = 0.001).

### 3.2. Analysis of Patients

#### 3.2.1. Clinical Features

There were 12,897 male patients in this group, accounting for 81.00%, which was 4.26 times that of female patients and was similar to that in Europe and America. The average age was (X34.97 ± 18.23) years old. The majority of patients were young adults aged 31 to 65 (49.21%), the oldest was 93 years old and the youngest was 5 days old. Preschool children aged 0–6 accounted for 12.27%. The injury time was the lowest in February (5.63%) and the highest in April (10.16%). The city's manufacturing industry is relatively developed, and the injured environment is mainly indoors such as factories, workshops, shopping malls, and service places (61.98%). The left hand was injured more than the right (1.16 : 1), and the left finger was injured more than the right finger (1.2 : 1). The thumb was injured the most (27%), followed by the middle finger (24%) and the ring finger (22%). The most common types of injuries were crush injury (34.16%) and cutting injury (29.44%). The average hospital stay of the patients in this group was 11.22 ± 12.75 days, and 79.41% of the patients were hospitalized for 1 to 2 weeks. The clinical cure rate was 5.67%, and the improvement rate was 94.28%. Eight patients were discharged from the hospital without healing due to concurrent infection, necrosis of the replanted finger, and nonunion of severed finger fractures.

#### 3.2.2. Occupational Injuries

Hand trauma is a common surgical disease, and with the development of industrial machinery production, the incidence of acute occupational hand trauma is increasing [20]. Occupational injuries accounted for 68.25% of the cases in this group, of which the ratio of male to female was 5.23 : 1. There were more people aged 20–50 (74.18%), and men and young adults were the high-risk groups for occupational hand trauma. The main types of injuries were crush injury (36.11%), cutting injury (29.92%), and fracture (9.40%).

#### 3.2.3. Injuries of Preschool Children

In this group, preschool children accounted for 12.27%, and the male: female ratio was 1.96 : 1, of which the 2–3-year-old group accounted for the highest proportion (45.70%). The main cause of injury was finger breakage (36.85%), followed by finger crush injury (16.63%) and cutting injury (15.15%).

### 3.3. Precautions

#### 3.3.1. Improve Manufacturing Intelligence

In recent years, with the rapid development of IT in China and the rapid popularization of AI, occupational hand injuries have been gradually decreased. However, still the development is unbalanced. Some of the larger industrial and mining enterprises have a low level of mechanical intelligence, insufficient protective facilities in the workplace, and the incidence of occupational injuries remains high. Therefore, it is recommended that the regulatory authorities improve the transformation and upgrading of equipment in large industrial and mining enterprises. At the same time, increase the promotion and application of AI technology, further improve the intelligence of the manufacturing industry, strictly enforce the access system, and reduce occupational injuries.

#### 3.3.2. Improve the Comprehensive Quality of the Whole People

According to research in [21], there is a correlation between occupational hand trauma and risk factors such as contact with machine failure, change in operation method, sudden discomfort, lack of protective equipment, use of sharp knives, unfamiliar tools, unfamiliar machines, change of job, temporary overtime, temporary rush to work, and sudden physical discomfort during the dangerous period (30 minutes prior to the injury). The risk factors of occupational injury are closely related to many factors such as gender, age, occupation, cultural literacy, safety awareness, operational proficiency, working environment, conditions, and psychological status. It is recommended to strengthen vocational education, improve professional quality, strengthen safety awareness, improve the working environment, strengthen humanistic care, care about the physical and mental health of workers, and prevent safety accidents.

#### 3.3.3. Strengthen the Supervision of Infants and Young Children

Traumatic injury of infants and young children has become one of the major problems in the social and public health fields of our country. To carry out in-depth clinical research on hand trauma in infants and young children, understand its clinical characteristics, strengthen preventive measures, reduce its incidence, and reduce the disability rate, should be raised as a systematic project to care for people's livelihood in today's society. This study showed that the proportion of hand trauma in preschool children was higher (12.27%), and the 2–3-year-old group was the main injury target of preschool children (45.70%). Because during this period, children appear as appearances, and it is also a period when their desire to imitate is high, showing the buds of exploration and knowledge, and they will make some inexplicable actions because of a strong need for independence, so it is easy to cause injury. Therefore, this stage of children is also an extraordinary period of parental supervision. In addition, the number of hand injuries in infants and young children is increasing year by year, which is also directly related to the increase of left-behind children in rural my country, or the fact that parents bring their children to work in cities, the ability to monitor infants and young children is insufficient, or they directly bring infants and young children into the work area [22]. Therefore, government care, social support, and the strengthening of guardianship are important measures to reduce accidental injury to infants and young children.

## 4. Conclusion

According to this study, males are more likely than females to sustain hand trauma; occupational injuries are more common than living injuries; males between the ages of 20 and 50 are at an increased risk of suffering an occupational injury. The incidence rate is highest in April every year and lowest in February. It mainly occurs in construction sites and factory workshops. The main types of injuries are crush injuries and cutting injuries. The main types of injuries were crush injury (36.11%), cutting injury (29.92%), and fracture (9.40%). Accidental injury of preschool children is also a social problem that cannot be ignored. This study showed that the proportion of hand trauma in preschool children was higher (12.27%), and the 2–3-year-old group was the main injury target of preschool children (45.70%). Therefore, measures such as improving the working environment, increasing the intelligence of the manufacturing industry, strengthening safety awareness, strengthening government functions, improving the quality of the whole people, strengthening humanistic care, and increasing infant care will greatly reduce the prevalence of hand trauma. To further extension of this research study, soft computing approaches can be adapted to predict the hand trauma injuries in the occupational environment [23, 24].

## Figures and Tables

**Table 1:** Distribution of injured environment (%).

Classify	Interior	Outside	Total	Statistical analysis
Occupational	6209 (39.00)	4657 (29.25)	10866 (68.25)	*t* = 6.999, *p* = 0.090
Living injury	3660 (22.99)	1396 (8.77)	5056 (31.75)	*t* = 2.231, *p* = 0.268
Total	9869 (61.98)	6053 (38.02)	15922 (100)	

**Table 2:** Distribution of injured sites (%).

Classify	Construction site	Workshop	Road transport	Service place	Sports place	Office space	Leisure place	Field work	Other	Total	Statistical analysis
Occupational	3622	4242	622	1217	104	518	202	105	234	10866	*t* = 2.273,
											*P* = 0.053
Living injury	0	0	958	843	489	868	869	768	261	5056	*t* = 4.347,
											*P* = 0.002
Total	3622	4242	1580	2060	593	1386	1071	873	495	15922	
(%)	22.75	26.64	9.92	12.94	3.72	8.70	6.73	5.48	3.11	100	

**Table 3:** Distribution of injury types.

Classify	Detached injury	Crushed injury	Concis	Frustration crack	Puncture injury	Fracture	Burn scald	Other	Total	Statistical analysis
Occupational	976	3924	3251	201	626	1021	198	669	10866	*t* = 2.701,
										*P* = 0.031
Living injury	401	1515	1436	162	705	501	157	179	5056	*t* = 3.212,
										*P* = 0015
Total	1377	5439	4687	363	1331	1522	355	848	15922	
(%)	8.65	34.16	29.44	2.28	8.36	9.56	2.23	5.33	100	

## Data Availability

The data used to support the study are included in the paper.
